# Coordinate regulation of fibronectin matrix assembly by the plasminogen activator system and vitronectin in human osteosarcoma cells

**DOI:** 10.1186/1475-2867-6-8

**Published:** 2006-03-28

**Authors:** Daniel Vial, Elizabeth Monaghan-Benson, Paula J McKeown-Longo

**Affiliations:** 1 Center for Cell Biology and Cancer Research, MC-165, Albany Medical College, 47 New Scotland Avenue, Albany, New York 12208, USA

## Abstract

**Background:**

Plasminogen activators are known to play a key role in the remodeling of bone matrix which occurs during tumor progression, bone metastasis and bone growth. Dysfunctional remodeling of bone matrix gives rise to the osteoblastic and osteolytic lesions seen in association with metastatic cancers. The molecular mechanisms responsible for the development of these lesions are not well understood. Studies were undertaken to address the role of the plasminogen activator system in the regulation of fibronectin matrix assembly in the osteoblast-like cell line, MG-63.

**Results:**

Treatment of MG-63 cells with P25, a peptide ligand for uPAR, resulted in an increase in assembly of fibronectin matrix which was associated with an increase in the number of activated β1 integrins on the cell surface. Overexpression of uPAR in MG-63 cells increased the effect of P25 on fibronectin matrix assembly and β_1 _integrin activation. P25 had no effect on uPAR null fibroblasts, confirming a role for uPAR in this process. The addition of plasminogen activator inhibitor Type I (PAI-1) to cells increased the P25-induced fibronectin polymerization, as well as the number of activated integrins. This positive regulation of PAI-1 on fibronectin assembly was independent of PAI-1's anti-proteinase activity, but acted through PAI-1 binding to the somatomedin B domain of vitronectin.

**Conclusion:**

These results indicate that vitronectin modulates fibronectin matrix assembly in osteosarcoma cells through a novel mechanism involving cross-talk through the plasminogen activator system.

## Introduction

Bone metastasis is a significant complication in tumor progression and a contributing cause of patient mortality. Contributing factors to such skeletal "homing" include the ability of the tumor to stimulate remodeling of the bone matrix thereby providing a niche supportive of its survival and growth [reviewed in [1]]. Fibronectin is a component of the bone matrix and is necessary for osteoblast differentiation and survival [2-4]. Recent in vivo studies have demonstrated a role for specific matrix-remodeling proteases (e.g. plasminogen activators) in the maintenance of bone mass and tissue composition [5], suggesting both fibronectin and the plasminogen activator system are important regulators of bone formation.

Formation of fibronectin matrix requires a cell-driven mechanical stretching of fibronectin, which is progressively incorporated into a dense detergent-insoluble fibrillar network via interactions with other cell-associated fibronectin dimers [reviewed in [6]]. The assembly of the fibronectin matrix requires activated α5β1 integrins [7,8]. Regulation of α_5_β_1 _integrin activation is thought to involve changes in integrin conformation which affect its affinity for fibronectin [9]. Downstream of fibronectin ligation, additional integrin dependent steps in the regulation of matrix assembly may occur in response to changes in key intracellular signaling pathways [10] or through the formation of complexes with either cytoskeletal proteins [11] or cell surface molecules including the glycosylphosphatidylinositol (GPI)-anchored urokinase-type plasminogen activator receptor (uPAR) [12-14]. High levels of uPAR expression have been shown to lead to formation of uPAR/β1 complexes which modulate integrin signaling and adhesive function [15,16]. uPAR effects on α5β1 function are complex and may result in either gain or loss of function depending on cellular context [15,17]. Several peptides have been identified which can modulate the functional association of integrins with uPAR [12,18]. Among them, peptide P25, isolated from a peptide library screening, is able to directly bind to uPAR and modulate integrin activity [12,15,19,20].

uPAR binds directly to the somatomedin B (SMB) domain of vitronectin and supports adhesion of a number of different cell types [21,22]. Plasminogen activator inhibitor Type I (PAI-1), the major physiological regulator of uPA activity, binds in close proximity to the uPAR recognition site within the SMB domain and can competitively inhibit or displace uPAR-vitronectin interactions [22,23]. The PAI-1 binding site on vitronectin is close to the only RGD motif in vitronectin [21] and the binding of PAI-1 to vitronectin can sterically inhibit integrin dependent adhesion to this motif [22,24]. Thus, PAI-1 can modulate the association of both uPAR- and/or integrins with vitronectin.

Treatment of human dermal fibroblasts with the uPAR ligand, P25, results in a marked increase in the polymerization of the fibronectin matrix and in the level of β1 integrin activation [13,14]. In the present study, we now show that overexpression of uPAR in MG-63 cells increases the effects of P25 on both fibronectin matrix assembly as well as integrin activation. In addition, P25 has no effect on matrix assembly in uPAR null cells. Our results also show that uPAR and PAI-1 synergistically regulate the amount of fibronectin deposition into the matrix. The positive regulation of PAI-1 on fibronectin assembly requires the presence of vitronectin suggesting that PAI-1 and vitronectin work cooperatively to regulate the amount of fibronectin matrix in MG-63 cells.

## Results

### The uPAR ligand P25 enhances the β1 integrin-dependent formation of fibronectin matrix in MG-63 cells

**Figure 1:**
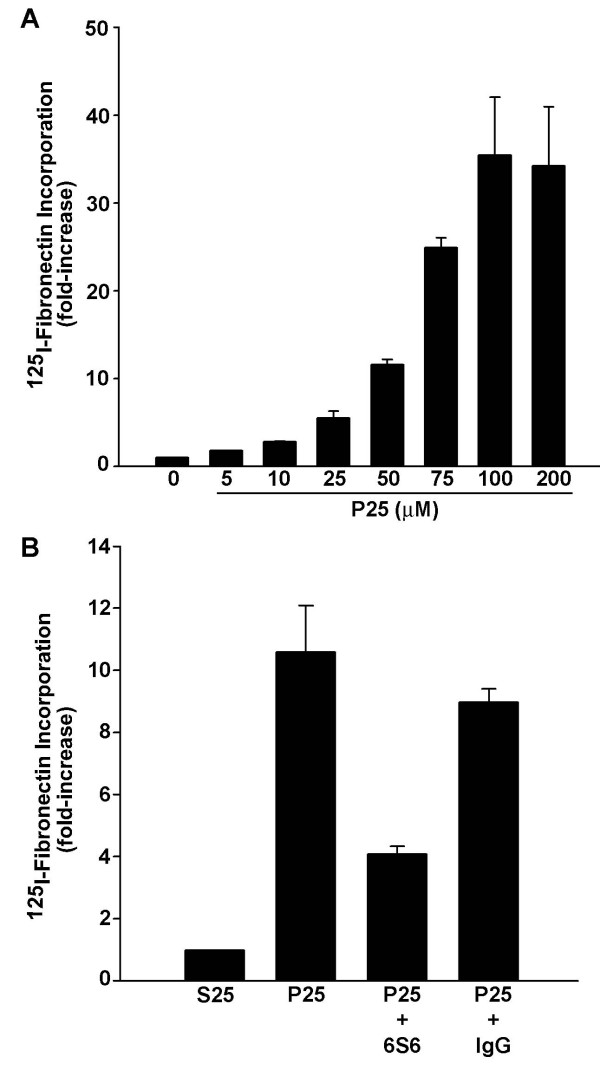
**uPAR regulation of fibronectin matrix formation in MG-63 cells**. **A **– MG-63 cells were incubated with ^125^I-fibronectin for 6 hours in the presence of the indicated concentrations of P25 in DMEM containing 0.02% BSA. Cell layers were extracted with 1% DOC, and ^125^I-fibronectin that was incorporated into the detergent-insoluble matrix fibronectin was recovered by centrifugation and measured by gamma scintillation. Control wells contained no added peptide. **B **– MG-63 cells were preincubated for 2 h with 20 μg/ml of normal mouse IgG or clone 6S6 antibody, a function-blocking monoclonal antibody against the β1 integrin, and then treated with 50 μM P25 or S25 in the presence of ^125^I-fibronectin (FN) for 6 hours. Cell layers were extracted in 1% DOC, and ^125^I-Fn that was incorporated into the detergent-insoluble matrix was recovered by centrifugation and measured by gamma scintillation.

We have previously shown that uPAR can regulate fibronectin matrix assembly in human fibroblast cells [14]. To determine whether a similar regulation existed in bone cells, human osteosarcoma (MG-63) cell layers were incubated with the uPAR ligand P25. Matrix assembly was assessed by monitoring the incorporation of ^125^I-fibronectin into detergent insoluble matrix. Incubation of osteosarcoma cells with P25 resulted in a dose-dependent increase in the assembly of ^125^I-fibronectin into the detergent-insoluble extracellular matrix (Figure 1A). Concentrations of P25 ranging from 10 to 200 μM produced a 3–35-fold increase in the level of fibronectin present in the detergent-insoluble matrix. Effects of P25 on matrix assembly were saturable reaching a maximum increase at doses between 75–100 μM. Experiments done in the presence of the control scrambled peptide, S25, were similar to untreated cell layers and indicated that S25 had no effect on matrix assembly (data not shown). The P25 enhancement of matrix assembly was largely dependent on the β_1 _integrin, as a function-blocking monoclonal antibody against the β_1 _integrin, clone 6S6 [34,35], attenuated the effects of P25 on matrix assembly by 65% (Figure 1B).

### P25 enhances fibronectin deposition into preformed matrix and accelerates the formation of extracellular (ECM) contacts

**Figure 2:**
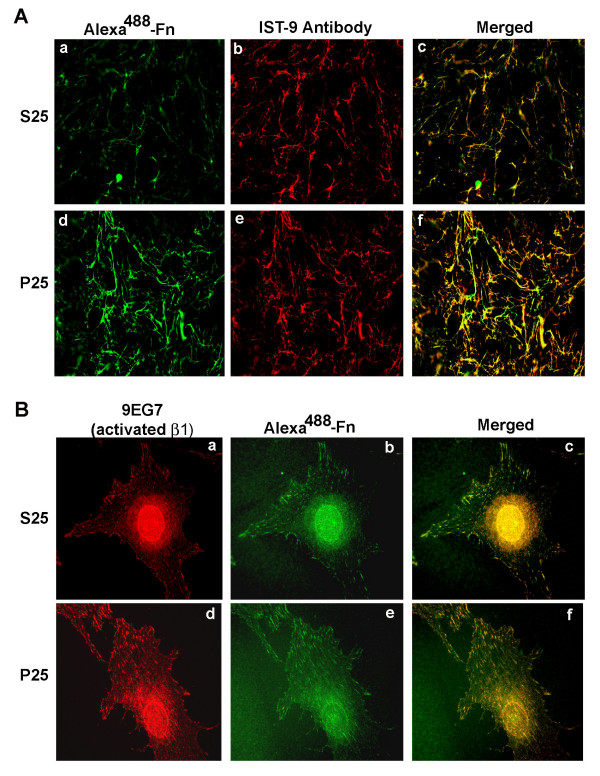
**Increase in the formation of matrix and ECM contacts in P25-treated MG-63 cells**. **A **– MG-63 cell monolayers were incubated for 16 hours in DMEM containing 2 μg/ml AlexaFluor^488 ^derivatized plasma fibronectin (green) in the presence of 50 μM S25 (a, b) or P25 (d, e). Cells were subsequently fixed, permeabilized and endogenous fibronectin was visualized by indirect immunofluorescence using the IST-9 antibody specific for cell-derived fibronectin (red). Panels c and f show merged images indicating colocalization of P25-dependent fibronectin polymerization with endogenous fibronectin matrix. **B **– Cells in suspension were allowed to attach for 6 hours on fibronectin-coated coverslips in DMEM containing soluble AlexaFluor^488^-fibronectin (20 μg/ml, green), in the presence of 50 μM S25 (a, b) or P25 (d, e). Cells were fixed, permeabilized, and activated β1 integrin was visualized by indirect immunofluorescence using the 9EG7 antibody (red). Panels c and f show merged images indicating colocalization of activated integrins with fibronectin fibers.

The localization of exogenous plasma fibronectin within the cell layer following P25 treatment was determined using fluorescence microscopy. Cell monolayers were incubated for 16 hours with 2 μg/ml plasma fibronectin derivatized with AlexaFluor^488 ^in the presence of 50 μM of either P25 or S25. The deposition of exogenous AlexaFluor^488 ^derivatized fibronectin into the extracellular matrix was greatly enhanced in the presence of P25 (Figure 2A). Endogenous fibronectin was visualized using the IST-9 monoclonal antibody, which recognizes the alternatively spliced type III repeat, EDA, not found in plasma fibronectin [36]. The merged images indicate extensive co-localization of exogenous fibronectin with the endogenous fibronectin matrix, indicating that the P25 stimulates the assembly of exogenous fibronectin into preformed matrix.

The effect of P25 on the initial stages of matrix assembly was also examined. The early stages of fibronectin matrix assembly have been shown to occur in regions of cell-extracellular matrix (ECM) contact [37,38]. To examine the role of uPAR in the formation of ECM contacts, cells in suspension were allowed to attach for 6 hours on fibronectin-coated coverslips in medium containing soluble AlexaFluor^488^-fibronectin in the presence of 50 μM S25 or P25 (Figure 2B). Areas of cell-ECM contact were visualized by staining for active β1 integrin using the antibody 9EG7 [32]. Control cells treated with S25 showed clustered active β1 integrins which colocalized with fine fibronectin fibrils (Figure 2B, a-c). Active integrins and fibronectin fibrils were primarily seen at the periphery of the cell. Incubation of MG-63 cells with P25 induced a clear enhancement in the number of ECM contacts with more clusters of active integrins seen in the central and perinuclear regions of the cell. These results indicate that P25 stimulates the formation of ECM contacts in MG-63 cells.

### uPAR levels modulate the effects of P25 on the assembly of fibronectin matrix

**Figure 3:**
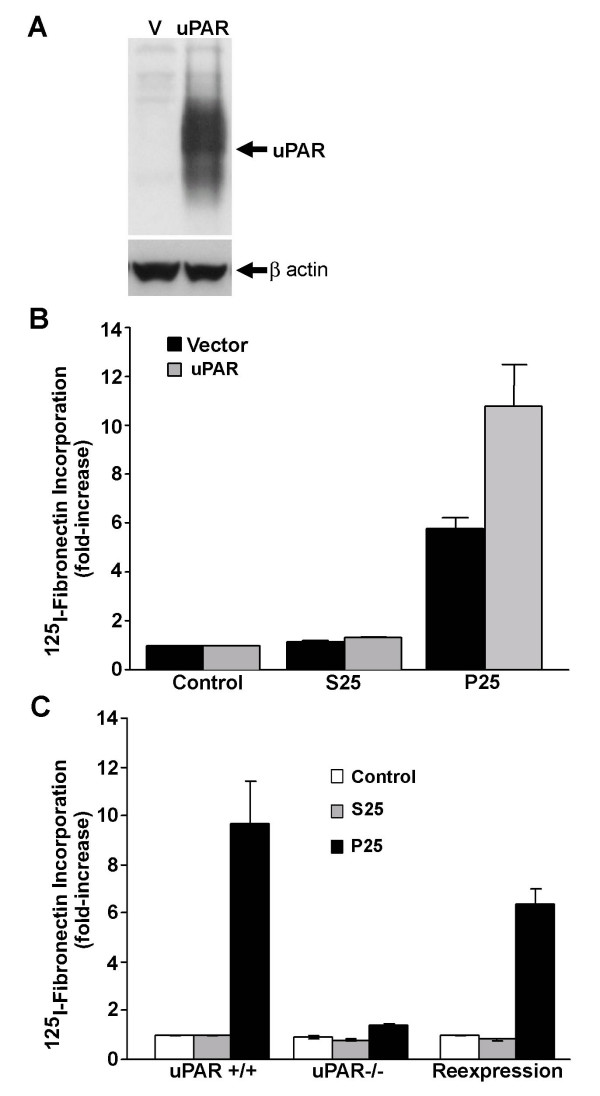
**Effect of uPAR expression levels on P25-induced fibronectin matrix assembly**. **A **– Lysates were prepared from vector (V) or uPAR transfected (uPAR) stable lines of MG-63 cells. uPAR levels were analyzed by Western blotting with anti-uPAR antibody. The membrane was stripped and reprobed with anti β-actin antibody as a loading control. **B **– Vector and uPAR transfected cell lines were cultured overnight and then stimulated with 50 μM S25 or P25 for 6 h in the presence of ^125^I-fibronectin. Cell layers were extracted in 1% DOC, and ^125^I-fibronectin that was incorporated into the detergent-insoluble matrix fibronectin was recovered by centrifugation and measured by gamma scintillation. **C **– uPAR-/- MEF were transiently transfected with the cDNA for human uPAR. 24 hours after transfection, MEFs were washed and monolayers were stimulated with 50 μM S25 or P25 for 6 hours in the presence of ^125^I-fibronectin. Cell layers were extracted in 1% DOC, and ^125^I-fibronectin that was incorporated into detergent-insoluble matrix was recovered by centrifugation and measured by gamma scintillation. Control cells received no added peptide.

To determine whether the effect of P25 on matrix assembly was specific for uPAR, experiments were designed to address whether the P25-mediated increase in fibronectin matrix assembly was sensitive to uPAR levels. MG-63 cells were stably transfected with a plasmid containing the cDNA for human uPAR. Parental MG-63 cells express uPAR but at reduced levels compared with either human fibrosarcoma (HT-1080) or human fibroblast cells (data not shown). MG-63 cells transfected with uPAR exhibited a large increase in the levels of uPAR as compared with cells transfected with vector (V) alone (Figure 3A). uPAR was not visible in the vector alone transfectants due to the exposure time of the blot; however, these cells exhibited the same level of endogenous uPAR as the parental MG-63 cells (data not shown). uPAR overexpressing cell lines were compared with cell lines transfected with vector alone for their ability to polymerize fibronectin (Figure 3B). When compared with cells transfected with only vector in the presence of control peptide S25, cell lines overexpressing uPAR exhibited no change in the assembly of fibronectin matrix, indicating that overexpression of uPAR did not affect basal levels of matrix assembly. When stimulated with the uPAR ligand, P25, cells expressing only vector exhibited a 6-fold increase in the level of matrix assembly. This stimulation by P25 was increased to nearly 12-fold in cells overexpressing uPAR.

To confirm a role for uPAR in P25-mediated fibronectin matrix assembly, fibroblasts derived from uPAR null mice were tested for their ability to assemble matrix in response to P25. As shown in Figure 3C, incubation of wild-type mouse embryo fibroblasts (MEF) with P25 resulted in a 10-fold increase in matrix assembly. In contrast, there was no effect of P25 on matrix assembly by uPAR-/- cells. Reexpression of uPAR in uPAR -/- cells restored the ability of the cells to increase matrix assembly in response to P25. There was no difference in the incorporation of fibronectin into matrix between the uPAR +/+ and uPAR -/- cells in the absence of P25. The data indicate that uPAR is required for the effects of P25 on matrix assembly and that the levels of uPAR on the cell surface can modulate the cellular response to P25. The data further indicate that basal levels of matrix assembly are not under uPAR regulation.

### P25 increases the level of active β1 integrins on uPAR overexpressing cells

**Figure 4:**
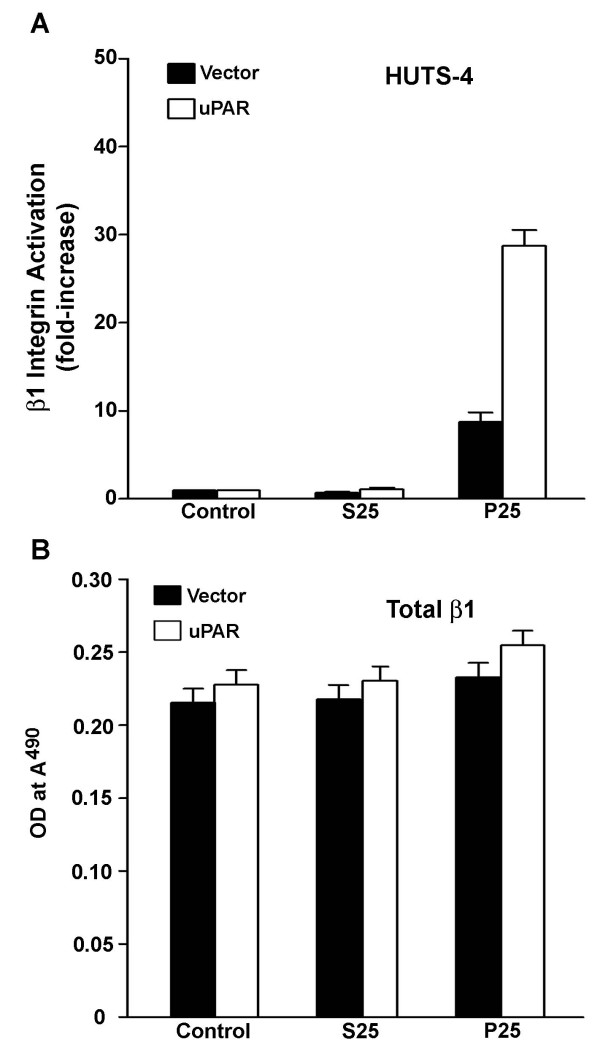
**Overexpression of uPAR increases P25-induced β1 integrin activation**. Stable MG-63 cell lines transfected with either vector alone or vector containing the cDNA for human uPAR were grown to confluency in 24-well plates. The following day, cells were incubated for 2 hours in DMEM containing 50 μM P25 or S25. Supernatant was removed and cells were then incubated for 1 hour with 100 ng/ml of a monoclonal antibody (HUTS-4) which recognized either the active form of the β1 integrin (**A**) or another monoclonal antibody which recognized total β1 integrin (**B**). Cell layers were washed, fixed in paraformaldehyde, and incubated with a peroxidase conjugated secondary antibody. Bound antibody was detected by incubating cells with substrate, and color development was measured at *A*_490 _in a plate reader.

To determine whether the enhanced effect of P25 on MG-63 cells overexpressing uPAR was associated with changes in integrin activation, the effect of P25 on β1 integrin activation was evaluated in MG-63 cells which overexpress uPAR. Activated integrins were detected using the monoclonal antibody HUTS-4, which recognizes only the active conformation of the β_1 _integrin. Overexpression of uPAR in untreated cells or control peptide, S25, treated cells had no effect on basal levels of HUTS-4 binding (Figure 4A), suggesting that overexpression of uPAR alone does not effect integrin activation or changes in matrix assembly in MG-63 cells. The addition of P25 to vector-transfected cells resulted in a 8–9 fold increase in the level of HUTS-4 binding as compared with S25-treated cells. In cell lines overexpressing uPAR, the addition of P25 led to a nearly 30-fold increase in HUTS-4 binding. This suggests that the over-expression of uPAR leads to a greater β_1 _integrin activation after P25 treatment, as compared to vector-transfected cells. To determine whether the addition of P25 or the overexpression of uPAR affected the total number of β_1 _integrins on the cell surface, cells overexpressing uPAR or cells containing vector alone were incubated with P25, and levels of β_1 _were assessed using P5D2, an antibody that recognizes active and inactive forms of β_1_. Neither P25 nor uPAR overexpression modified the surface expression of β_1 _integrin (Figure 4B). These data indicate that addition of P25 to MG-63 cells results in an increase in the activation state of the β_1 _integrin and that uPAR levels modulate the P25 response in these cells. Since the β_1 _integrin blocking antibody 6S6 reduced the P25-induced fibronectin matrix assembly (Figure 1B), our data also suggest that the effects of P25 on matrix assembly result from an increase in uPAR-dependent β_1 _integrin activation.

### Effect of uPA and PAI-1 on integrin activation and fibronectin matrix assembly

**Figure 5:**
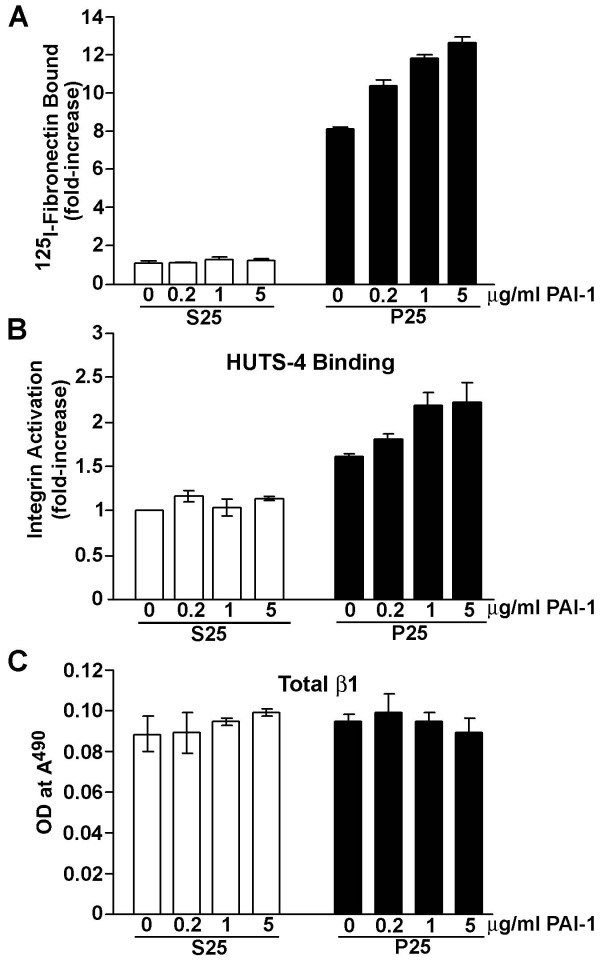
**Effect of PAI-1 on P25 induced integrin activation and matrix assembly**. **A **– MG-63 cells monolayers, grown to confluency in 12-well plates, were washed and pretreated with different concentrations of PAI-1 for 20 minutes in the presence of ^125^I-fibronectin and then stimulated with 50 μM S25 or P25 for 6 hours. Cells were rinsed, scraped directly into 1 ml of 1% deoxycholate and the total cell bound ^125^I-fibronectin was counted. **B, C **– MG-63 cells, grown to confluency in 24-well plates, were washed, pretreated with different concentrations of PAI-1 for 20 minutes and incubated for 1 hour in DMEM containing 50 μM P25 or S25. Cells were then incubated for 1 hour with 100 ng/ml of either monoclonal antibody, HUTS-4 (**B**) or P5D2 **(C)**. Cell layers were washed, fixed in paraformaldehyde, and incubated with a peroxidase conjugated secondary antibody. Bound antibody was detected by incubating cells with substrate, and color development was measured at *A*_490 _in a plate reader.

To determine whether other components of the plasminogen-activator system could affect the assembly of the fibronectin matrix, cells were incubated with increasing concentrations of urokinase-type plasminogen activator (uPA) or PAI-1. Incubation of MG-63 cells with increasing doses of active uPA had no effect on the assembly of fibronectin matrix in S25- and P25-treated MG-63 cells (data not shown). PAI-1, however, was able to modulate matrix assembly. Treatment of MG-63 cells with increasing doses of PAI-1 had no effect on fibronectin binding to the cell layer in the presence of the control peptide S25 (Figure 5A). In contrast, PAI-1 in the presence of the P25 peptide elicited a dose-dependent increase in fibronectin deposition. Maximum levels of fibronectin deposition were seen between 1–5 μg/ml PAI-1 as increasing amounts of PAI-1 resulted in no further stimulation (data not shown). The PAI-1 mediated increase in fibronectin binding to the cell layer was accompanied by an increase in β1 integrin activation (Figure 5B). PAI-1 had no effect on the level of total β1 integrin (Figure 5C). These data indicate that under conditions of uPAR stimulation, PAI-1 positively regulates both β1 integrin activation and matrix assembly.

### PAI-1 enhances P25-stimulated fibronectin polymerization through a vitronectin dependent mechanism

**Figure 6:**
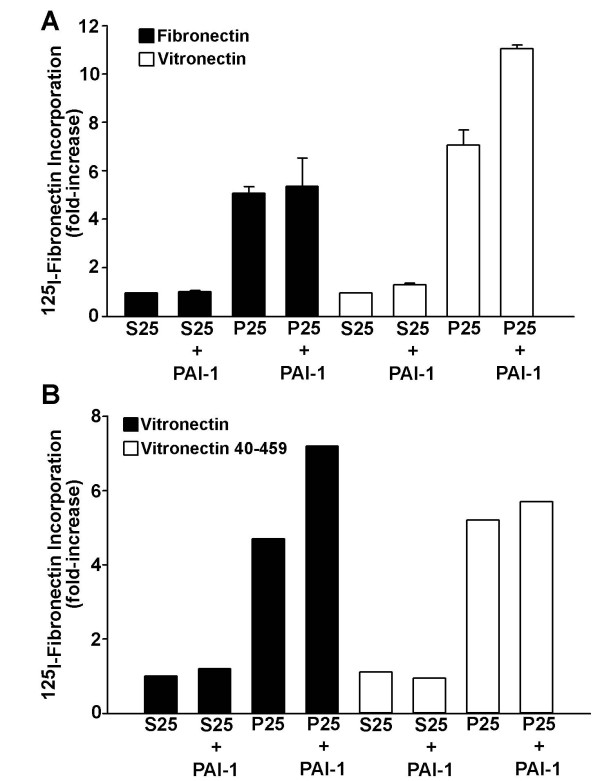
**PAI-1 enhancement of matrix assembly requires vitronectin**. **A **– MG-63 cells were plated overnight on fibronectin- or vitronectin-coated wells in serum-free media. Cells were then pretreated with PAI-1 (1μg/ml) for 20 minutes in the presence of ^125^I-fibronectin and 50 μM peptide was added for 6 hours. Cell layers were extracted in 1% DOC, and ^125^I-fibronectin that was incorporated into the detergent-insoluble matrix was recovered by centrifugation and measured by gamma scintillation. Control levels (S25-treated cells) of fibronectin incorporation were set at 1 for each substrate. **B **– MG-63 cells were incubated overnight on wells coated with vitronectin or vitronectin fragment 40–459 in serum-free media. Cells were then pretreated with 1 μg/ml wild-type PAI-1 for 20 minutes in the presence of ^125^I-fibronectin and 50 μM peptide was added for 6 hours. Cell layers were processed as described in **A.**

In addition to binding to uPA/uPAR complexes, PAI-1 also binds directly to matrix vitronectin. To determine whether the effects of PAI-1 on matrix assembly required vitronectin, MG-63 cells were seeded overnight onto wells coated with either fibronectin or vitronectin. PAI-1 had no effect on matrix assembly when cells were adherent to fibronectin, but stimulated the amount of fibronectin incorporated into the matrix when cells were plated onto vitronectin-coated substrates (Figure 6A). Several studies have shown that the PAI-1 binding site in vitronectin is within the amino terminal 40-amino acid somatomedin B (SMB) domain [21,23,39]. To evaluate whether the binding of PAI-1 to vitronectin was required for the effect of PAI-1 on matrix formation, cells were seeded overnight on wells coated with either vitronectin or the vitronectin fragment 40–459, which lacks the PAI-1 binding site. This vitronectin fragment contains a deletion of the SMB domain (aa 1–39), but retains the integrin binding site and mediates attachment and spreading of cells. The stimulatory effect of PAI-1 on the P25 induced formation of fibronectin matrix was not seen on cells plated onto wells coated with the vitronectin fragment 40–459 (Figure 6B). These results suggest that the regulation of fibronectin matrix polymerization by PAI-1 requires the binding of PAI-1 to vitronectin.

**Figure 7:**
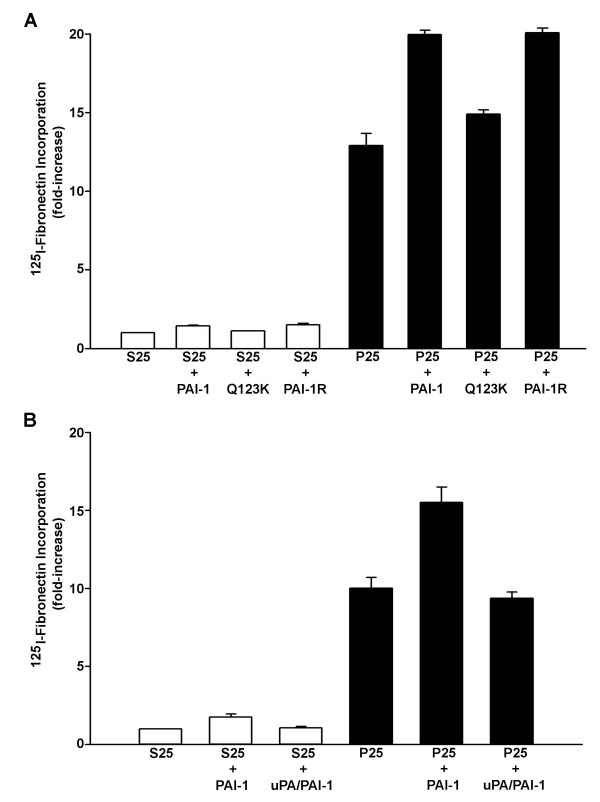
**Effect of PAI-1 mutants and uPA-PAI-1 complex on matrix formation**. **A **– MG-63 cells were incubated overnight on vitronectin-coated wells in serum-free media. Cells were then pretreated with 1 μg/ml (20 nM) wild-type PAI-1 or PAI-1 mutants (Q123K or PAI-1R) for 20 min in the presence of ^125^I-fibronectin and 50 μM peptide was added for 6 hours. Cell layers were extracted in 1% DOC, and ^125^I-fibronectin that was incorporated into the detergent-insoluble matrix was recovered by centrifugation and measured by gamma scintillation. B – Cells were pretreated with 20 nM wild-type PAI-1 or uPA-PAI-1 complex for 20 min in the presence of ^125^I-fibronectin and 50 μM peptide was added for 6 hours. Cell layers were processed as described in A.

In addition to binding to vitronectin, PAI-1 also binds to uPA and inhibits its catalytic activity. To determine whether PAI-1's vitronectin or uPA binding sites were required for PAI-1's effect on matrix assembly, mutant forms of PAI-1 were compared for their ability to stimulate matrix assembly on cells adherent to vitronectin (Figure 7A). PAI-1R, a mutant form which does not bind to uPA but does bind to vitronectin [25] stimulated fibronectin polymerization to the same extent as wild-type PAI-1, indicating that the effects of PAI-1 on matrix assembly do not require the association of PAI-1 with uPA. In contrast, PAI-1 (Q123K) which does not bind to vitronectin but does bind uPA [40], failed to enhance the P25-dependent matrix assembly. Similarly, no stimulatory effect on matrix formation was observed when MG-63 cells were treated with the preformed complex uPA-PAI, which is not able to bind vitronectin [41,42] (Figure 7B). Collectively, these results indicate that the stimulatory effect of PAI-1 on fibronectin matrix assembly requires PAI-1 binding to vitronectin but not uPA.

## Discussion

We have identified a role for the plasminogen activator system and vitronectin in the regulation of fibronectin matrix assembly in the human osteoblast-like cell line, MG-63. uPAR acts in concert with PAI-1 to stimulate fibronectin matrix deposition by increasing the level of α5β1 integrin activation. In addition, the effects of P25 on assembly of fibronectin matrix were modulated by the level of uPAR expression. Cells overexpressing uPAR were more sensitive to the effects of P25, while uPAR -/- cells were unable to respond to P25. Reexpression of uPAR in uPAR -/- cells restored the P25 response confirming that the effect of P25 on matrix assembly was specific to uPAR. This finding is in agreement with earlier studies which have shown that matrix assembly can be regulated through uPAR [14]. Other studies have shown that in some tumor cells, high levels of uPAR expression correlated with an increase in the formation of fibronectin matrix [43]. In our studies, neither increasing the levels of uPAR in MG-63 nor restoring uPAR to uPAR -/- cells increased the incorporation of fibronectin into matrix, unless the cells were stimulated with P25. This suggests that in MG-63 cells as well as MEFs, basal levels of matrix assembly are not under uPAR regulation.

P25 induced a several fold increase in the rate of assembly of exogenous fibronectin into previously established matrix. Furthermore, when suspended MG-63 cells were allowed to adhere to fibronectin, P25 increased the number and size of ECM contacts as well as the level of activated β1 integrin present in the ECM contacts. Several earlier studies have shown that polymerization of fibronectin matrix originates in cell adhesion sites, requires the presence of activated β1 integrins and can be modulated by changes in the level of integrin activation [7,8,37]. Taken together, these results suggest that in MG-63 cells ligation of uPAR with P25 results in an increase in both assembly of exogenous fibronectin into preformed matrix as well as an increase in the initiation of matrix assembly sites in newly adherent cells. The effects of P25 on matrix assembly were significantly attenuated in the presence of a blocking antibody to the β1 integrin, suggesting that the P25 induced increase in matrix assembly was the direct result of the increase in β1 integrin activation. This observation is in agreement with previous studies showing that addition of P25 to cells stimulates the adhesion function of the α5β1 integrin [15,19]. P25 induced matrix assembly was not completely inhibited by β1 blocking antibodies suggesting that P25 may stimulate alternate mechanisms of matrix assembly by promoting the binding of fibronectin to β3 integrins or to unfolded matrix fibronectin molecules [44,45].

P25 directly interacts with uPAR and shares some sequence homology with integrin α subunits [12,15,18]. uPAR has been found to both physically and functionally associate with several different integrin receptors [46,47]. The direct interaction of P25 with uPAR may well affect uPAR modulation of integrin function [12,48], however its mechanism of action remains unclear. Dimerization/oligomerization of uPAR can affect its subcellular localization by driving uPAR into lipid rafts [49]. As lipid rafts are sites of integrin activation [50], P25-mediated changes in uPAR localization to lipid rafts could promote the activation of raft-associated integrins.

PAI-1 may play a role in the regulation of the uPAR-dependent formation of fibronectin matrix. Up-regulation of matrix assembly by PAI-1 was observed under conditions of uPAR stimulation and was accompanied by an increase in the activation of the β1 integrin. The PAI-1 mediated increase in integrin activation and fibronectin matrix assembly required the binding of PAI-1 to vitronectin, but was independent of the binding of PAI-1 to uPA. The mechanism whereby PAI-1 binding to vitronectin might further increase β1 integrin activation is not known. Binding of PAI-1 to the SMB domain of vitronectin blocks the binding of both uPAR and vitronectin-integrin receptors to vitronectin [22,24], suggesting that loss of either uPAR-vitronectin and/or integrin-vitronectin binding may be part of the mechanism by which PAI-1 modulates fibronectin matrix formation. Vitronectin negatively regulates matrix assembly [51-53] therefore, "disengagement" of either uPAR or vitronectin integrins from vitronectin by PAI-1 may release the inhibitory effect of vitronectin on matrix assembly. Alternatively, PAI-1 mediated release of uPAR from vitronectin may free uPAR to form complexes with other cell surface molecules. Complexes of uPAR with α5β1 integrins would then promote further integrin activation [13,17]. Alternatively, uPAR may complex with other receptors including G-protein linked receptors or growth factor receptors which affect signaling molecules important in integrin function [12,48,54]. A recent study has reported that PAI-1 initiates cellular detachment from both vitronectin and fibronectin substrates, suggesting a PAI-dependent negative effect on the adhesive function of both β3 and β1 integrins [55]. In these studies, the effects of PAI-1 on cell adhesion were independent of vitronectin, but required the binding of PAI-1 to uPA. The reason for the discrepancy between the two studies may reflect the choice of cell type. Our experiments were performed on MG-63 cells which assemble a robust extracellular matrix, while the earlier studies used HT-1080 fibrosarcoma cells which do not assemble an extracellular matrix.

Bone destruction during metastasis is marked by loss of collagenous matrix [56]. Deposition and organization of collagenous matrix is intrinsically linked to the assembly of the fibronectin matrix which serves as the core scaffolding on which the collagenous matrix is built [57,58]. Our results in MG-63 cells highlight a new mechanism by which PAI-1 and uPAR work synergistically to increase the formation of fibronectin matrix and suggests that therapeutic antagonists of this pathway may attenuate the inappropriate deposition of excess matrix often seen in osteoblastic lesions. Alternatively, modalities directed at increasing levels of fibronectin in bone matrix (i.e., uPAR ligands) might prove useful in the control of osteolytic metastasis, particularly under conditions where inhibitors of bone resorption are not effective [59]. Increased levels of fibronectin, moreover, would be expected to promote the differentiation and survival of osteoblasts, which are often rendered inactive in sites of bone metastasis. Future studies are needed to determine whether the vitronectin/plasminogen activator system are therapeutic targets for the control of bone metastasis.

## Conclusion

Our data show that uPAR and PAI-1 can positively regulate the activation state of the β1 integrin and increase the rate of fibronectin polymerization in human osteosarcoma cells. The effects of PAI-1 on matrix assembly are independent of uPA but require the binding of PAI-1 to vitronectin. These results highlight a new role for vitronectin and the plasminogen activation system in the regulation of bone matrix remodeling.

## Methods

### Reagents and antibodies

Unless otherwise stated, all chemicals were purchased from Sigma (St. Louis, MO). The uPAR ligand, peptide P25, sequence AESTYHHLSLGYMYTLN, and the scrambled peptide, S25, sequence NYHYLESSMTALYTLGH, were synthesized by Cell Essentials (Boston, MA). The anti-β_1 _antibodies, clones P5D2, 6S6, and HUTS-4 were purchased from Chemicon (Temecula, CA). Control mouse IgG and antibody to β-actin were obtained from Sigma (St. Louis, MO). Secondary antibodies goat anti-mouse and goat anti-rabbit HRP were purchased from Bio-Rad (Hercules, CA). AlexaFluor^594^-labeled goat anti-rat antibody was obtained from Molecular Probes (Eugene, OR). 9EG7 antibody was obtained from Pharmingen (San Diego, CA) and IST-9 antibody from Abcam (Cambridge, England). The pCEP4 plasmid was from Invitrogen (Carlsbad, CA) and the pCEP4 plasmid containing the full length cDNA for human uPAR was described previously [15] and was the gift of Dr. H. Chapman (University of California-San Francisco). Recombinant active PAI-1, active uPA, and uPAR polyclonal antibody (399R) were from American Diagnostica (Greenwich, CT). The non-vitronectin binding PAI-1 mutant, Q123K, was from Molecular Innovations (Southfield, MI) and the dual PAI-1R (T333→R and A335→R) which resulted in the loss of the protease inhibitory activity of PAI-1 but did not affect its binding affinity for vitronectin, was kindly provided by Dr. D. Lawrence (Holland Lab, American Red Cross, Rockville, MD) [25]. The fragment of vitronectin 40–459, which lacks the somatomedin B domain, was the gift of Dr R.P. Czekay (Albany Medical College, Albany, NY), and was expressed and purified as described previously [21].

### Cell culture

The MG-63 osteosarcoma cells were grown in Dulbecco's modified Eagle's medium (DMEM, Invitrogen), containing antibiotics (penicillin-streptomycin) and 10% fetal bovine serum (FBS, Hyclone Laboratories, Logan, UT). The uPAR+/+ and uPAR-/- mouse embryo fibroblasts (MEF) cell lines have been described previously [26] and were provided by Dr. Steve Gonias (University of California-San Diego). MEF were cultured in DMEM containing 10% FBS, plus antibiotics.

### Isolation of the uPAR overexpressing MG-63 cell lines

Two μg of the expression vector (pCEP4) or the pCEP4-uPAR plasmid were transfected into MG-63 cells (3 × 10^5^) using Lipofectamine 2000 (Invitrogen). The following day, cells were trypsinized and transferred into two 100 mm diameter tissue culture plates. Selection (100 μg/ml hygromycin) was started 48 hours after the transfection. Clones were identified, isolated with cloning rings, and expanded for testing. All transfected cell lines were screened for uPAR expression by Western blotting. Three clones overexpressing uPAR and three clones containing only vector were selected for expansion and further characterization.

### Transient transfection of uPAR in MEF

uPAR-/- MEFs were transfected with 4 μg of pcDNA 3.1+ plasmid containing the full length cDNA for human uPAR (pcDNA-uPAR) and 12 μl LipofectAMINE^TM^, according to the manufacturer instructions (Invitrogen, Carlsbad, CA). Control cells were transfected with empty vector. Transfection efficiencies were approximately 60%, as determined by counting fluorescent cells co-transfected with pEGFP plasmid (Clontech). MEF were transfected for 24 hours before matrix incorporation assay. Cells transfected with empty vector (pLDNA3.1+) served as controls.

### Purification of fibronectin and vitronectin

Human plasma fibronectin was purified from a fibronectin- and fibrinogen-rich by-product of Factor VIII production by ion exchange chromatography on DEAE-cellulose (Amersham Biosciences) as described previously [27]. Vitronectin was purified from fibronectin- and fibrinogen-depleted human plasma by heparin-Sepharose affinity chromatography according to the method of Yatohgo et al. [28]. Purified plasma fibronectin (400 μg) was iodinated with 1 mCi of Na^125^I (Perkin Elmer Life Sciences) as described previously [29]. Iodinated fibronectin was mixed with bovine albumin, 1 mg/ml, dialyzed against phosphate-buffered saline, and frozen at -80°C until used. Fibronectin (2 mg/ml) was derivatized with AlexaFluor^488 ^according to the manufacturer's protocol (Molecular Probes).

### Matrix incorporation assay

MG-63 cells were seeded onto 6 well plates (2.5 × 10^5 ^cells/well) in complete medium. The following day, cultures were incubated for 6 hours with ^125^I-fibronectin (2 μg/ml; 1 × 10^6 ^cpm/ml) in DMEM plus 0.02% BSA in the presence of the uPAR ligand, P25, or the control peptide, S25. For isolation of detergent insoluble matrix, cells were rinsed in PBS, extracted in 1% deoxycholate (DOC) (in 20 mM Tris (pH 8.8) buffer containing 2 mM phenylmethylsulfonyl fluoride, 2 mM EDTA, 2 mM N-ethylmaleimide, and 2 mM iodoacetic acid). Detergent insoluble matrix was obtained by centrifugation at 18,000 rpm for 40 min and associated radioactivity present in the pellet measured using gamma scintillation. Preincubation with 20 nM PAI-1 (active or mutant) or uPA/PAI-1 complex and ^125^I-fibronectin (2 μg/ml; 1 × 10^6 ^cpm/ml) was for 20 min in DMEM+BSA 0.02% before peptide addition. The uPA/PAI-1 complex was prepared by reacting active two chain uPA with an equimolar concentration of PAI-1 for 10 min at 37°C as described previously [30]. In some experiments, cells were pre-incubated with blocking antibody to the β1 integrin prior to incubation with ^125^I-fibronectin. To determine the total cell layer-associated fibronectin, cell cultures were pretreated for 20 min with different concentrations of PAI-1 or uPA with ^125^I-fibronectin (2 μg/ml; 1 × 10^6 ^cpm/ml) in DMEM at 37 °C and then stimulated with either P25 or S25 for 6 hours. After incubation, cells were rinsed three times in PBS, then scraped directly into 1 ml of 1% deoxycholate and the total cell bound ^125^I-fibronectin was counted. In some experiments, tissue culture wells were precoated with either 10 μg/ml fibronectin, vitronectin or vitronectin fragment 40–459 in PBS for 2 hours at 37°C.

### Fluorescent microscopy

To localize the P25-dependent plasma fibronectin deposited into matrix with endogenous cell-derived fibronectin, MG-63 cells were cultured in complete medium for 24 hours. Cell monolayers were incubated for an additional 16 hours in serum-free medium containing 50 μM S25 or P25 and plasma fibronectin (2 μg/ml) derivatized with AlexaFluor^488^. Cell layers were then rinsed, fixed with 3% paraformaldehyde, permeabilized with 0.2% Triton for 5 min, and blocked for 1 hour with 1% BSA in PBS. Endogenous cell-derived fibronectin was visualized by incubating cells with IST-9 monoclonal antibody (2.5 μg/ml) against cellular fibronectin. This antibody recognizes fibronectin containing the EDA domain which is found exclusively in cell-derived fibronectin. Cells were rinsed and incubated with goat anti-mouse AlexaFluor^594^-labeled secondary antibody. To examine the formation of ECM contacts, coverslips were coated with fibronectin (10 μg/ml), and blocked in 1% BSA. MG-63 cells were allowed to attach for 6 hours on fibronectin-coated coverslips in DMEM containing soluble AlexaFluor^488^-fibronectin (20 μg/ml) and peptide (P25 or S25: 50 μM). Cells were washed, fixed in 3% paraformaldehyde, permeabilized with 0.2% Triton for 5 min and blocked with 3% milk. Cells were then incubated for 1 hour with 5 μg/ml 9EG7 antibody against active β_1 _integrin and bound antibody visualized using AlexaFluor^594^-conjugated goat anti-rat antibody. Fluorophores were visualized using an Olympus BMX-60 microscope equipped with a cooled LCD sensi-camera. Images were acquired using Slidebook Software (Intelligent Imaging Innovation, Inc., Denver, CO) and processed using Adobe Photoshop.

### Immunoblotting

Cell layers (3 × 10^5^/well) were washed with PBS and lysed with hot sample buffer [75 mM Tris-HCl (pH 6.8), 2% SDS, 10% glycerol]. The cell lysates were boiled for 2 min and the proteins were separated by SDS-PAGE (Bio-Rad, Hercules, CA), transferred to nitrocellulose membranes and blotted with the indicated antibodies.

### Integrin activation assay

MG-63 cell lines were grown overnight (6 × 10^4 ^cells/well) in 24-well plates in complete medium. The following day, cells were treated for 2 hours in DMEM containing either P25 or S25 and subsequently incubated for 1 hour at 37°C with either 100 ng/ml of HUTS-4 antibody, which recognizes the activated conformation of the β_1 _integrin [31-33], or 100 ng/ml P5D2, an antibody that recognizes all forms of β_1 _integrin. Cells were rinsed with PBS, fixed with 3% paraformaldehyde, blocked in 3% BSA, and incubated for 1 hour with HRP-conjugated goat anti-mouse antibody. Freshly prepared substrate (0.1 M citrate buffer, 0.5 mg/ml *o*-phenyl-enediamine, 1 μl/ml 30% hydrogen peroxide pH 5.0) was added to each well, and the color was allowed to develop. The reaction was stopped with the addition of 2 N sulfuric acid, and the OD was measured at *A*_490_. Measurements were corrected for light scattering by subtracting the OD obtained at *A*_650_. In some experiments, cells were incubated overnight on either fibronectin or vitronectin-coated plates in DMEM containing 0.02% BSA and preincubated for 20 min with PAI-1 (1 μg/ml) before treatment with peptides.

## Competing interests

The author(s) declare that they have no competing interests.

## Authors' contributions

DV designed and carried out most of the experiments and drafted the manuscript. EM-B participated in performing and designing several experiments. PM-L conceived the study, coordinated the experimental questions, and prepared the final version of the manuscript. All authors read and approved the final manuscript.

## References

[B1] Edlund M, Sung S-Y, Chung LWK (2004). Modulation of prostate cancer growth in bone microenvironments. J Cell Biochem.

[B2] Moursi AM, Damsky CH, Lull J, Zimmerman D, Doty SB, Aota S (1996). Fibronectin regulates calvarial osteoblast differentiation. J Cell Sci.

[B3] Globus RK, Doty SB, Lull JC, Holmuhamedov E, Humphries MJ, Damsky CH (1998). Fibronectin is a survival factor for differentiated osteoblasts. J Cell Sci.

[B4] Globus RK, Moursi A, Zimmerman D, Lull J, Damsky C (1995). Integrin-extracellular matrix interactions in connective tissue remodeling and osteoblast differentiation. ASGSB Bull.

[B5] Daci E, Everts V, Torrekens S, Van Herck E, Tigchelaar-Gutterr W, Bouillon R (2003). Increased bone formation in mice lacking plasminogen activators. J Bone Miner Res.

[B6] Wierzbicka-Patynowski I, Schwarzbauer JE (2003). The ins and outs of fibronectin matrix assembly. J Cell Sci.

[B7] Wu C, McDonald JA, Keivens VM, O'Toole TE, Ginsberg MH (1995). Integrin activation and cytoskeletal interaction are essential for the assembly of a fibronectin matrix. Cell.

[B8] Sechler JL, Corbett SA, Schwarzbauer JE (1997). Modulatory roles for integrin activation and the synergy site of fibronectin during matrix assembly. Molec Biol Cell.

[B9] Mould AP, Askari JA, Barton S, Kline AD, McEwan PA, Craig SE (2002). Integrin activation involves a conformational change in the alpha 1 helix of the beta subunit A-domain. J Biol Chem.

[B10] Brenner KA, Corbett SA, Schwarzbauer J (2000). Regulation of fibronectin matrix assembly by activated Ras in transformed cells. Oncogene.

[B11] Giannone G, Jiang G, Sutton DH, Critchley DR, Sheetz MP (2003). Talin1 is critical for force-dependent reinforcement of initial integrin-cytoskeleton bonds but not tyrosine kinase activation. J Cell Biol.

[B12] Wei Y, Eble JA, Wang Z, Kreidberg JA, Chapman HA (2001). Urokinase receptors promote b1 integrin function through interactions with integrin a3b1. Mol Biol Cell.

[B13] Aguirre-Ghiso JA, Liu D, Mignatti A, Kovalski K, Ossowski L (2001). Urokinase receptor and fibronectin regulate the ERK(MAPK) to p38(MAPK) activity ratios that determine carcinoma cell proliferation or dormancy in vivo. Mol Biol Cell.

[B14] Monaghan E, Gueorguiev V, Wilkins-Port C, McKeown-Longo PJ (2004). The receptor for urokinase-type plasminogen activator regulates fibronectin matrix assembly in human skin fibroblasts. J Biol Chem.

[B15] Wei Y, Lukashev M, Simon DI, Bodary SC, Rosenberg S, Doyle MV (1996). Regulation of integrin function by the urokinase receptor. Science.

[B16] Aguirre-Ghiso JA, Kovalski K, Ossowski L (1999). Tumor dormancy induced by down-regulation of urokinase receptor in human carcinoma involves integrin and MAPK signaling. J Cell Biol.

[B17] Wei Y, Yang X, Liu Q, Wilkins JA, Chapman HA (1999). A role for caveolin and the urokinase receptor in integrin-mediated adhesion and signaling. J Cell Biol.

[B18] Simon DI, Weis Y, Zhang L, Rao NK, Xu H, Chen Z (2000). Identification of a urokinase receptor-integrin interaction site. J Biol Chem.

[B19] van der Pluijm G, Sijmons B, Vloedgraven H, vander Bent C, Drijfhout JW, Verheijen J (2001). Urokinase-receptor/integrin complexes are functionally involved in adhesion and progression of human breast cancer in vivo. Am J Pathol.

[B20] Ahmed N, Oliva K, Wang Y, Quinn M, Rice G (2003). Downregulation of urokinase plasminogen activator receptor expression inhibits Erk signalling with concomitant suppression of invasiveness due to loss of uPAR-b1 integrin complex in colon cancer cells. Brit J Cancer.

[B21] Okumura Y, Kamikubo Y, Curriden SA, Wang J, Kiwada T, Futaki S (2002). Kinetic analysis of the interaction between vitronectin and the urokinase receptor. J Biol Chem.

[B22] Deng G, Curriden SA, Hu G, Czekay RP, Loskutoff DJ (2001). Plasminogen activator inhibitor-1 regulates cell adhesion by binding to the somatomedin B domain of vitronectin. J Cell Physiol.

[B23] Deng G, Curriden SA, Wang S, Rosenberg S, Loskutoff DJ (1996). Is plasminogen activator inhibitor-1 the molecular switch that governs urokinase receptor-mediated cell adhesion and release?. J Cell Biol.

[B24] Stefansson S, Lawrence DA (1996). The serpin PAI-1 inhibits cell migration by blocking integrin a_v_b_3 _binding to vitronectin. Nature.

[B25] Stefansson S, Petitclerc E, Wong MK, McMahon GA, Brooks PC, Lawrence DA (2001). Inhibition of angiogenesis in vivo by plasminogen activator inhibitor-1. J Biol Chem.

[B26] Ma Z, Thomas KS, Webb DJ, Moravec RSAM, Mars WM, Gonias SL (2002). Regulation of Rac1 activation by the low density lipoprotein receptor-related protein. J Cell Biol.

[B27] Zheng M, McKeown-Longo PJ (2002). Regulation of HEF1 expression and phosphorylation by TGF-b1 and cell adhesion. J Biol Chem.

[B28] Yatohgo T, Izumi M, Kashiwagi H, Hayashi M (1988). Novel purification of vitronectin from human plasma by heparin affinity chromatography. Cell Struct Funct.

[B29] McKeown-Longo PJ, Mosher DF (1983). Binding of plasma fibronectin to cell layers of human skin fibroblasts. J Cell Biol.

[B30] Webb DJ, Thomas KS, Gonias SL (2001). Plasminogen activator inhibitor 1 functions as a urokinase response modifier at the level of cell signaling and thereby promotes MCF-7 cell growth. J Cell Biol.

[B31] Luque A, Gomez M, Puzon W, Takada Y, Sanchez-Madrid F, Cabanas C (1996). Activated conformations of very late activation integrins detected by a group of antibodies (HUTS) specific for a novel regulatory region (355–425) of the common b1 chain. J Biol Chem.

[B32] Lenter M, Uhlig H, Hamann A, Jeno P, Imhof B, Vestweber D (1993). A monoclonal antibody against an activation epitope on mouse integrin b1 blocks adhesion of lymphocytes to the endothelial integrin a6b1. Proc Natl Acad Sci USA.

[B33] Mould AP, Barton SJ, Askari JA, McEwan PA, Buckley PA, Craig SE (2003). Conformational changes in the integrin bA domain provide a mechanism for signal transduction via hybrid domain movement. J Biol Chem.

[B34] Gao JX, Wilkins J, Issekutz AC (1995). Migration of human polymorphonuclear leukocytes through a synovial fibroblast barrier is mediated by both b2 (CD11/CD18) integrins and the b1 (CD29) integrins VLA-5 and VLA-6. Cell Immunol.

[B35] Wilkins JA, Li A, Ni H, Stupack DG, Shen C (1996). Control of b1 integrin function. Localization of stimulatory epitopes. J Biol Chem.

[B36] Carnemolla B, Borsi L, Zardi L, Owens RJ, Baralle FE (1987). Localization of the cellular-fibronectin specific epitope recognized by the monoclonal antibody IST-9 using fusion proteins expressed in E. coli. FEBS Lett.

[B37] Christopher RA, Kowalczyk AP, McKeown-Longo PJ (1997). Localization of fibronectin matrix assembly sites on fibroblasts and endothelial cells. J Cell Sci.

[B38] Pankov R, Cukierman E, Katz BZ, Matsumoto K, Lin DC, Lin S (2000). Integrin dynamics and matrix assembly: tensin-dependent translocation of a5b1 integrins promotes early fibronectin fibrillogenesis. J Cell Biol.

[B39] Seiffert D, Ciambrone G, Wagner NV, Binder BR, Loskutoff DJ (1994). The somatomedin B domain of vitronectin. J Biol Chem.

[B40] Lawrence DA, Berkenpas MB, Palaniappan S, Ginsburg D (1994). Localization of vitronectin binding domain in plasminogen activator inhibitor-1. J Biol Chem.

[B41] Declerck PJ, De Mol M, Alessi M-C, Baudner S, Paques E-P, Preissner KT (1988). Purification and characterization of a plasminogen activator inhibitor 1 binding protein from human plasma. Identification as a multimeric form of S-protein (vitronectin). J Biol Chem.

[B42] Arroyo De Prada N, Schroeck F, Sinner E-K, Muehlenweg B, Twellmeyer J, Sperl S (2002). Interaction of plasminogen activator inhibitor type-1 (PAI-1) with vitronectin. Eur J Biochem.

[B43] Aguirre-Ghiso JA, Estrada Y, Liu D, Ossowski L (2003). ERK(MAPK) activity as a determinant of tumor growth and dormancy; regulation by p38(SAPK). Cancer Res.

[B44] Zoppi N, Gardella R, De Paepe ABS, Colombi M (2004). Human fibroblasts with mutations in *COL5A1 *and *COL3A1 *genes do not organize collagens and fibronectin in the extracellular matrix, down-regulate a_2_b_1 _integrin, and recruit a_v_b_3 _instead of a_5_b_1_integrin. J Biol Chem.

[B45] Ohashi T, Erickson HP (2005). Domain unfolding plays a role in superfibronectin formation. J Biol Chem.

[B46] Blasi F, Carmeliet P (2002). uPAR: a versatile signalling orchestrator. Nat Rev Mol Cell Biol.

[B47] Kugler MC, Wei Y, Chapman HA (2003). Urokinase receptor and integrin interactions. Curr Pharm Des.

[B48] Liu D, Aguirre-Ghiso JA, Estrada Y, Ossowski L (2002). EGFR is a transducer of the urokinase receptor initiated signal that is required for in vivo growth of a human carcinoma. Cancer Cell.

[B49] Cunningham O, Andolfo A, Santovito ML, Luzzolino L, Blasi F, Sidenius N (2003). Dimerization controls the rapid raft partitioning of uPAR/CD87 and regulates its biological functions. The EMBO J.

[B50] Decker L, Ffrench-Constant C (2004). Lipid rafts and integrin activation regulate oligo-dendrocyte survival. J Neurosci.

[B51] Bae E, Sakai T, Mosher DF (2004). Assembly of exogenous fibronectin by fibronectin-null cells is dependent on the adhesive substrate. J Biol Chem.

[B52] Hocking DC, Sottile J, Reho T, Fassler R, McKeown-Longo PJ (1999). Inhibition of fibronectin matrix assembly by the heparin-binding domain of vitronectin. J Biol Chem.

[B53] Zhang Q, Sakai T, Nowlen J, Hayashi I, Fassler R, Mosher DF (1999). Functional b1-integrins release the suppression of fibronectin matrix assembly by vitronectin. J Biol Chem.

[B54] Resnati M, Pallavicini I, Wang JM, Oppenheim J, Serhan CN, Romano M (2002). The fibrinolytic receptor for urokinase activates the G protein-coupled chemotactic receptor FPRL1/LXA4R. PNAS.

[B55] Czekay R-P, Aertgeerts K, Curriden SA, Loskutoff D (2003). Plasminogen activator inhibitor-1 detaches cells from extracellular matrices by inactivating integrins. J Cell Biol.

[B56] Costa L, Demers LM, Gouveia-Oliveira A, Schaller J, Costa EB, de Moura MC (2002). Prospective evaluation of the peptide-bound collagen type I cross-links N-telopeptide and C-telopeptide in predicting bone metastases. J Clin Oncol.

[B57] Velling T, Risteli J, Wennerberg K, Mosher DF, Johansson S (2002). Polymerization of type I and III collagens is dependent on fibronectin and enhanced by integrins α11β1 and α2β1. J Biol Chem.

[B58] Sottile J, Hocking DC (2002). Fibronectin polymerization regulates the composition and stability of extracellular matrix fibrils and cell-matrix adhesions. Molec Biol Cell.

[B59] Lee YP, Schwarz EM, Davies M, Jo M, Gates J, Zhang X (2002). Use of zoledronate to treat osteoblastic versus osteolytic lesions in a severe-combined-immunodeficient mouse model. Cancer Res.

